# Conflict Data Fusion in a Multi-Agent System Premised on the Base Basic Probability Assignment and Evidence Distance

**DOI:** 10.3390/e23070820

**Published:** 2021-06-28

**Authors:** Jingyu Liu, Yongchuan Tang

**Affiliations:** School of Big Data and Software Engineering, Chongqing University, Chongqing 401331, China; 20184423@cqu.edu.cn

**Keywords:** Dempster–Shafer evidence theory, uncertainty, multi-agent information fusion, base basic probability assignment

## Abstract

The multi-agent information fusion (MAIF) system can alleviate the limitations of a single expert system in dealing with complex situations, as it allows multiple agents to cooperate in order to solve problems in complex environments. Dempster–Shafer (D-S) evidence theory has important applications in multi-source data fusion, pattern recognition, and other fields. However, the traditional Dempster combination rules may produce counterintuitive results when dealing with highly conflicting data. A conflict data fusion method in a multi-agent system based on the base basic probability assignment (bBPA) and evidence distance is proposed in this paper. Firstly, the new bBPA and reconstructed BPA are used to construct the initial belief degree of each agent. Then, the information volume of each evidence group is obtained by calculating the evidence distance so as to modify the reliability and obtain more reasonable evidence. Lastly, the final evidence is fused with the Dempster combination rule to obtain the result. Numerical examples show the effectiveness and availability of the proposed method, which improves the accuracy of the identification process of the MAIF system.

## 1. Introduction

With the increasing popularity of artificial intelligence, agent technology has become a hot topic in the field of distributed artificial intelligence. Agents with autonomy and collaboration can deal with complex, collaborative, and unpredictable problems. They can modify their goals with changes in the environment, expand their knowledge, and improve their ability. Due to the constraints of ability and relationships with other agents, it is impossible to solve complex problems using a single agent. Artificial intelligence is maturing. It is committed to solving more complex, more realistic, and larger scale problems. These problems are beyond the capability of a single agent. Therefore, the multi-agent system (MAS) [1] has emerged as an important tool. It is a collection of agents, and its purpose is to solve the problems of large-scale, complex, real-time, and uncertain information. Its theoretical research value lies in two aspects: one is the development of a closed and isolated knowledge system into a distributed and open intelligent knowledge system, and the other is the development of a centralized intelligent system into a non-independent distributed intelligent system. The key point of MAS research is to enable independent agents to complete complex control tasks or solve complex problems through negotiation, coordination, and cooperation. However, uncertainty is the biggest challenge for MAS research. In many problems, the state of the environment will be uncertain due to the limitation of noise or sensor capacity. An agent can only observe the state of the environment through its own sensors. Therefore, the ability of an agent to predict the trend of other agents is limited. As a result, cooperation becomes complicated, and, therefore, conflicting information may appear in MAS.

Information fusion [2] is an application field that combines data from multiple sources to support decision analysis. Applying information fusion technology to MAS can process the information and provide more complete judgment, evaluation, and decision making. Using the appropriate information fusion method, the local information perceived by an agent is fused in space, time, and function. Therefore, identifying the way by which to fuse conflicting information and make correct judgments represents the main challenge in multi-agent information fusion (MAIF).

As an uncertain reasoning method, Dempster–Shafer evidence theory (D-S theory) has a strong ability to express and process uncertain information. It serves as a powerful tool in the representation and fusion of decision making with uncertain information. It has been widely used in the fields of information fusion [3], target recognition [4,5], risk analysis [6], classification [7,8,9], and decision making [10,11,12].

### 1.1. Motivation

In modern engineering applications, electronic information systems tend to be highly integrated, multi-component, and complex functions. Therefore, concurrency, suddenness, and complexity are the three major problems that may occur when the equipment fails [13,14,15]. In many information systems, multi-source information systems occupy a certain proportion, and they are usually used to represent complex information from multiple sources [16]. However, in the process of information fusion and diagnosis, many scholars have begun to focus on identifying methods of effectively integrating multi-source information [3] and measuring its uncertainty to ensure correctness and anti-interference [17,18].

The MAIF system mainly studies the communication, coordination, and conflict resolution between multiple agents. It is an autonomous solution to the problem of information fusion among agents in MAS. It focuses on the analysis of information fusion between multiple agents rather than the autonomy and development of a single agent. In the process of MAIF, even if all agents use the same original detection data, the conclusions given may be inconsistent, because the reasoning model used by each agent is not necessarily the same. There have been many attempts to improve performance, such as distributed weighting [19] and relative reliability evaluation [20,21]. However, they do not focus on the measurement of uncertainty between information sources from different institutions. Moreover, the methods somewhat struggle to combine conclusions in the process of MAIF.

D-S theory is a good choice for uncertain information processing in MAIF. However, D-S theory may fail in highly conflicting situations, which makes it difficult to guarantee the fusion result. This may lead to unreasonable results [22]. Two methods to improve the performance of MAIF are proposed: modifying the fusion rules and preprocessing the uncertain information before data fusion. Previous studies have proposed preprocessing strategies. They provided distance measurement to effectively measure the uncertainty of MAIF. However, the measurement object is mainly based on multiple data sources as a whole, and there is no specific distinction between the two data sources. Therefore, this paper proposes a new method for better data preprocessing. In this study, we add a new base basic probability assignment(nbBPA) based on base basic probability assignment (bBPA) [23,24] and reconstructed BPA [25] to preprocess the data, and we combine it with evidence distance to prove its effectiveness in the application of MAIF system.

### 1.2. Contributions

The main contributions of this method are different from those of a large number of existing methods and are summarized as follows:

- It is feasible and extensible to integrate evidence theory into the MAIF system. However, D-S theory may result in counterintuitive results in highly conflicting situations. In order to address D-S conflicting information fusion in MAIF, the method based on nbBPA and evidence distance is proposed.
- In the improved MAIF method, nbBPA provides a kind of prior information to preprocess the data. The evidence distance is used to judge the difference between the bodies of evidence. The weight of each belief function is calculated according to the evidence distance so as to recalculate the revised BPA.

### 1.3. Organization

The following remainder this paper are organized as follows: Section 2 investigates previous work related to this study. In Section 3, we review some basic concepts. Then, in Section 4, we propose a new preprocessing method, nbBPA, which is based on bBPA, and we propose an improved method using nbBPA. In Section 5, we show the performance of our proposed method based on three numerical examples. Finally, conclusions of the proposed method are given in Section 6.

## 2. Related Work

D-S theory was first proposed by Dempster in 1967 [26] and then further extended by Shafer [27]. Due to its strong ability to express and process uncertain information, the theory is widely used in information fusion systems, such as target recognition and remote sensing [28]. Although the application of D-S theory has made some progress, the D-S combination rule often obtains results contrary to common sense when it fuse highly conflicting evidence [29]. In view of these paradoxes, scholars have proposed some solutions that can be divided into two categories: one is based on modifying the D-S combination rule [30,31,32]; the other is based on modifying evidence sources [33,34]. The methods of relevant evidence fusion based on the modified evidence source can be divided into two types: one is based on the correlation source evidence model [35], and the other is based on the discount correction model.

The perspective of the discount correction model is the overestimation of the composite result. Because the D-S combination rule is used to fuse the relevant evidence directly, the relevant parts of the evidence are repeatedly calculated. The basic idea of this kind of method is that the relevant evidence should be discounted. The discount coefficient depends on the degree of correlation. Compared with independent evidence, relevant evidence has overlapping information that cannot be given the same weight in the fusion process.

Yager proposed a weighted fusion method of relevant evidence with the relative independence degree set as weight [36]. The so-called degree of relative independence refers to the degree of independence of the latter evidence relative to all previous evidence. The evidence uses the relative independence degree as a means of discounting the factors to process. In doing so, it can be regarded as independent of the previous evidence, and, thus, D-S combination rule can be used for fusion. The method depends on the order of evidence fusion. The evidence in the front has a great impact on the fusion results. Therefore, a method based on the information quantity of evidence is proposed in [36] to reduce the loss of information as much as possible. However, this method does not provide the basis for determining the degree of relative independence. When the amount of evidence is large, the computational complexity of the method greatly increases. In order to determine the correlation degree of the discount model, a method to solve the correlation discount coefficient based on the network analysis method is proposed in [37]. This method needs to rely on expert opinion for modeling, which has certain subjectivity.

Tessem [38] designed the pignistic probability distance to describe evidence similarity based on pignistic probability. The distance was used to evaluate the effectiveness of the approximate calculation algorithm of evidence combination. Bauer [39] also conducted a similar study. Pignistic probability distance has been widely used. However, there remain misunderstandings regarding its definition, and it is misused. Liu et al. [40] indirectly defined evidence distance based on DSMP probability. Cuzzolin [41] designed and proposed a geometric interpretation of evidence theory. On this basis, Jousselme et al. [42] defined the evidence distance. Then, many studies of evidence distance applications emerged. Deng et al. [43] defined the similarity between evidence volume using Jousselme evidence distance, and then the weight correction evidence to be combined was generated. Liu [44] used pignistic probability distance and Dempster combination conflict coefficient K to form a binary, which was used to describe the conflict between evidence bodies. Ristic et al. [45] realized target identity association based on multiple uncertain information sources in a TBM framework by using evidence distance. Zouhal et al. [46] introduced a mean square deviation distance based on pignistic probability, which corresponds to BPA. The distance effectively improved the accuracy rate of evidence of the k-nearest neighbor classifier. Schubert [47] used evidence distance for clustering analysis, and an ideal clustering performance was observed.

In addition, in order to solve the conflicting data in uncertain information, some methods attempt to assign initial belief to events in the frame of discernment (FOD). A new strategy to consider the initial belief degree of propositions in the power set of FOD is proposed in [23], but it may lead to decentralized belief allocation. An improved base basic probability assignment approach is proposed in [24]. These methods can introduce additional information to uncertain information modeling and processing.

## 3. Background

### 3.1. Dempster–Shafer Evidence Theory

#### 3.1.1. Framework of Discernment

Assuming that there is a problem to judge, all possible solutions N identified in this problem are described as a set. N are mutually exclusive and exhaustive. The set is also known as the framework of discernment (FOD). The FOD is shown as follows:

(1) $$\Theta \;=\;\;\;\;\{\;\;\;\theta_{1},\theta_{2},\cdots ,\theta_{n}\}$$

Its power set is

(2) $$2^{\Theta }=\left\{\begin{array}{c} \emptyset ,\left\{\theta_{1}\right\},\left\{\theta_{2}\right\},\ldots ,\left\{\theta_{N}\right\},\left\{\theta_{1},\theta_{2}\right\},\\ \ldots ,\left\{\theta_{1},\theta_{2},\ldots ,\theta_{i}\right\},\ldots ,\Omega \end{array}\right\}.$$

#### 3.1.2. Basic Probability Assignment

D-S theory assigns a probability to each possible solution within the framework of discernment (FOD), which is called basic probability assignment (BPA). The corresponding assignment function is called the mass function. BPA satisfies the following conditions:

(3) $$m\left(\emptyset \right)\;=\;0,\sum_{A\in \Theta }m\left(A\right)\;=\;1.$$

where *m* is the mass function of the FOD $2^{\Theta }$ and m(A) is the BPA value of proposition *A*, which represents how strongly the evidence supports the proposition *A*. If *m*(*A*) > 0, A is called a focal element.

#### 3.1.3. D-S Combination Rule

The D-S combination rule is a key step in synthesizing the information produced by multiple hypotheses. Two independent mass functions, $m_{1}$ and $m_{2}$, can be combined with the D-S combination rule as follows:

(4) $$m\left(A\right)\;=\;\left(m_{1}⊕m_{2}\right)\left(A\right)\;=\frac{1}{1-k}\sum_{B\cap C=A}m_{1}\left(B\right)m_{2}\left(C\right),$$

where $k\;=\sum_{B\cap C=\emptyset }m_{1}\left(B\right)m_{2}\left(C\right)$ where *k* represents the conflict degree between two bodies of evidence. If *k* = 0, $m_{1}$ and $m_{2}$ have no conflict. If *k* = 1, $m_{1}$ and $m_{2}$ are in complete conflict.

### 3.2. Base Basic Probability Assignment

The D-S combination rule may result in counterintuitive results when data with high conflict are fused. Thus, the bBPA is proposed to modify the BPA before data fusion.

#### 3.2.1. Base Basic Probability Assignment

Let Ω be a group of N mutually exclusive possible hypotheses. The power set of Ω is $2^{\Omega }$ in which the number of elements is $2^{N}$. If the FOD is complete, $m\left(\emptyset \right)\;=\;0$, then the base belief function $m_{b}$ is defined as follows:

(5) $$m_{b}\left(A_{i}\right)=\frac{1}{2^{N}-1}$$

where $A_{i}$ is the subset in Ω, except for the empty set ∅.

The initial belief allocated between basic events can introduce prior probability information to elements. In the unknown case, the average distribution of belief maximizes the entropy. The maximum entropy principle shows that when the entropy is maximum, the possible loss is small.

#### 3.2.2. Use the bBPA to Modify Initial BPA

bBPA is combined with the initial BPA to modify the BPA before data fusion, and it is defined as follows:

(6) $$m^{\prime }\left(A_{i}\right)=\frac{m_{b}\left(A_{i}\right)+m\left(A_{i}\right)}{2}$$

where $A_{i}$ is the subset in Ω, except for the empty set ∅.

### 3.3. Evidence Distance

In order to resolve the problem that the D-S combination rule produces, namely, counterintuitive results when fusing conflicting data, a previous study proposed a theory of evidence distance to measure the difference between two bodies of evidence. Then, some studies calculated the weight of each belief function based on the evidence distance so as to recalculate the modified BPA.

#### 3.3.1. Jousselme Evidence Distance

Let $m_{1}$ and $m_{2}$ be two BPAs on the same FOD Θ. Θ contains *N* mutually exclusive and exhaustive hypotheses. The distance between $m_{1}$ and $m_{2}$ is

(7) $$d_{BPA}\left(m_{i},m_{j}\right)=\sqrt{\frac{1}{2}{\left(\vec{m_{i}}-\vec{m_{j}}\right)}^{T}D\left(\vec{m_{i}}-\vec{m_{j}}\right)}$$

where *D* is an $2^{N}\times 2^{N}$ matrix whose elements are $D(A,B)=\frac{\left|A⋂B\right|}{\left|A⋃B\right|}$, A,B∈P(x)

From the previous definition, another way to write $d_{BPA}$ is as follows:

(8) $$d_{BPA}\left(m_{i},m_{j}\right)=\sqrt{\frac{1}{2}\left({∥\vec{m_{i}}∥}^{2}+{∥\vec{m_{j}}∥}^{2}-2<\vec{m_{i}},\vec{m_{j}}>\right)}$$

(9) $$<\vec{m_{i}},\vec{m_{j}}>=\sum_{i=1}^{2^{\theta }}\sum_{j=1}^{2^{\theta }}m_{1}(A_{s})m_{2}\left(B_{t}\right)\frac{\left|A_{s}⋂B_{t}\right|}{\left|A_{s}⋃B_{t}\right|}$$

where $A_{s}$,$B_{t}$ are the subsets of the FOD. For $d_{BPA}\in \left[0,1\right]$, the greater the value of $d_{BPA}$, the greater the difference between the two bodies of evidence.

#### 3.3.2. Combined Belief Function Based on Evidence Distance

In general, if a body of evidence is well supported by other bodies of evidence, the evidence is more important in the final fusion. On the contrary, if one piece of evidence conflicts with other bodies of evidence, it should have a smaller proportion in the final fusion. That is, the more similar one piece of evidence is to another, the higher its proportion in the final fusion.

The similarity between two bodies of evidence is denoted as $sim(m_{i},m_{j})$, which is defined as

(10) $$sim\left(m_{i},m_{j}\right)=1-d_{BPA}\left(m_{i},m_{j}\right)$$

Supposing the number of bodies of evidence is *k*, we can construct a similarity measure matrix (SMM) by obtaining all of the degrees of similarity between the bodies of evidence; $s_{ij}$ is short for $Sim(m_{i},m_{j})$.

(11) $$SMM=\left[\begin{array}{cccccc} 1 & s_{12} & \cdots & s_{1j} & \cdots & s_{1k}\\ \vdots & \vdots & \vdots & \vdots & \vdots & \vdots \\ s_{i1} & s_{i2} & \cdots & s_{ij} & \cdots & s_{ik}\\ \vdots & \vdots & \vdots & \vdots & \vdots & \vdots \\ s_{k1} & s_{k2} & \cdots & s_{kj} & \cdots & 1 \end{array}\right]$$

The support degree of a body of evidence $m_{i}$ (*i* = 1,2, *…*, *k*) is defined as

(12) $$Sup\left(m_{i}\right)=\sum_{j=1,j\neq i}^{k}s_{ij}=\sum_{j=1,j\neq i}^{k}sim(m_{i},m_{j})$$

The credibility degree $Crd_{i}$ of the body of evidence is defined as

(13) $$Crd_{i}=\frac{Sup(m_{i})}{\sum_{i=1}^{k}Sup(m_{i})}$$

It is clear that $\sum_{i=1}^{k}Crd_{i}=1$. Thus, the credibility degree is a weight that reflects the relative importance of the evidence collected. If a body of evidence is well supported by other bodies of evidence, its credibility degree is high. On the contrary, if one evidence body conflicts with other bodies of evidence, its credibility is low. After defining the credibility degree, the modified average of evidence (*MAE*) is given by assigning the weight:

(14) $$MAE\left(m\right)=\sum_{i=1}^{k}\left(Crd_{i}\times m_{i}\right)$$

If there are n bodies of evidence, one can use the classical D-S combination rule to combine the weighted average of the masses n-1 times.

### 3.4. Reconstructed BPA

The uncertainty of the recognition set is related to the number of elements it contains. The more elements a recognition set contains, the more uncertain its information will be, which corresponds to higher uncertainty. In order to gradually reduce the uncertainty, a reconstructed BPA $m_{r}$ is proposed [25]. It builds the relationship of a supporting degree between sets and its supporting source, not only from its own set but also from the sets that contain it. It is defined as follows:

(15) $$\left\{\begin{array}{cc} m_{r}\left(A_{i}\right)=\sum_{A_{i}\subseteq A_{j}}\frac{m(A_{j})}{2^{k}-1} & \forall A_{i},A_{j}\subset \Theta ,m\left(A_{i}\right)\neq 0\\ m_{r}\left(\Theta \right)=\frac{m(\Theta )}{2^{m}-1} & \end{array}\right.$$

where $A_{i}$,$A_{j}$ are the subsets in the FOD. $A_{i}$ set can be composed by either a single element or multiple elements. *K* is the number of elements corresponding to the set $A_{j}$. In order to meet the format requirements of the mass function in the revised D-S theory, it is necessary to normalize the reconstructed BPA. The sum of all $m_{r}$ results is used as a denominator, and normalization operation is as follows:

(16) $$m^{\prime }\left(A_{i}\right)=\frac{m_{r}\left(A_{i}\right)}{\sum_{i}^{2^{\Theta }}m_{r}\left(A_{i}\right)}$$

## 4. Multi-Agent Information Fusion

### 4.1. New Base BPA Definitions

bBPA is proposed for uncertain information processing, which equally distributes the probabilities to each BPA [23]. Although entropy can be maximized and the conflicting evidence can be fused more effectively without any initial conditions using this method, it also increases the uncertainty under certain conditions. The mass function of single-element subsets is effective in decision making, because in practical application, each mutually exclusive event is independent of each other. There can only be one element at a time, and multiple subset events represent the uncertainty of different elements. Therefore, increasing the value of single-element subsets allows for clearer results. Rather than assigning initial belief to the whole power set space, nbBPA is carried out for the single-element subsets. It is based on the proportion of the single-element subset in all of the mass functions. The initial belief assigned among the basic events can introduce prior probability information to the element. The nbBPA is defined as follows:

(17) $$\left\{\begin{array}{cc} m_{b}\left(A_{i}\right)=\frac{Mx(A_{i})}{n} & A_{i}\;\mathrm{is}\;a\;\mathrm{single}\;\mathrm{subset}\;\mathrm{event}\\ m_{b}\left(A_{i}\right)=0 & \mathrm{other} \end{array}\right.$$

where $Mx(A_{i})$ is the group number of mass functions, where $A_{i}$ is the highest in its single element subsets, and *n* is the group number of the mass functions where at least one single element is a focal element. The following is an example of calculating the nbBPA. For FOD Θ={A,B,C}, the BPAs are as follows:

$$\begin{array}{cccc} m_{1}\left(a\right)=0.8 & m_{1}\left(b\right)=0.2 & & \\ m_{2}\left(a\right)=0.3 & m_{2}\left(b\right)=0.5 & m_{2}\left(c\right)=0.2 & \\ m_{3}\left(a,b\right)=0.5 & m_{3}\left(a,b,c\right)=0.5 & & \\ m_{4}\left(a\right)=0.6 & m_{4}\left(b\right)=0.1 & m_{4}\left(c\right)=0.1 & m_{4}\left(a,b\right)=0.2\\ m_{5}\left(a\right)=0.4 & m_{5}\left(a,b,c\right)=0.6 & & \end{array}$$

In $m_{1}$,$m_{2}$,$m_{4}$ and $m_{5}$, at least one single element is a focal element, so *n* = 4.
In $m_{1}$, $m_{4}$ and $m_{5}$, a is the highest in the single-element subsets, so *M*x(*a*) = 3.
In $m_{2}$, b is the highest in the single-element subsets, so *M*x(*b*) = 1.
Thus, $m_{b}$(*a*) = 0.75, $m_{b}$(*b*) = 0.25.
$m_{b}$(*c*) = $m_{b}$(*a*,*b*) = $m_{b}$(*b*,*c*) = $m_{b}$(*a*,*c*) = $m_{b}$(*a*,*b*,*c*) = 0.

Then, $m_{b}$ is adopted to modify the initial BPA by calculating the arithmetic mean:

(18) $$\begin{array}{cc} m^{\prime }\left(A_{i}\right)=\frac{m_{b}\left(A_{i}\right)+m\left(A_{i}\right)}{2} & m(A_{i})\neq 0 \end{array}$$

where $A_{i}$ is the subset in the FOD. The $A_{i}$ set can consist of either a single element or multiple elements. To satisfy the format of mass function in evidence theory after correction, it is necessary to normalize the nbBPA. The sum of the calculation results is used as the denominator, and the operation of normalization is as follows:

(19) $$m^{\prime }\left(A_{i}\right)=\frac{m^{\prime }\left(A_{i}\right)}{\sum_{i}^{2^{\Theta }}m^{\prime }\left(A_{i}\right)}$$

### 4.2. Steps for Multi-Agent Information Fusion

**Figure 1:**
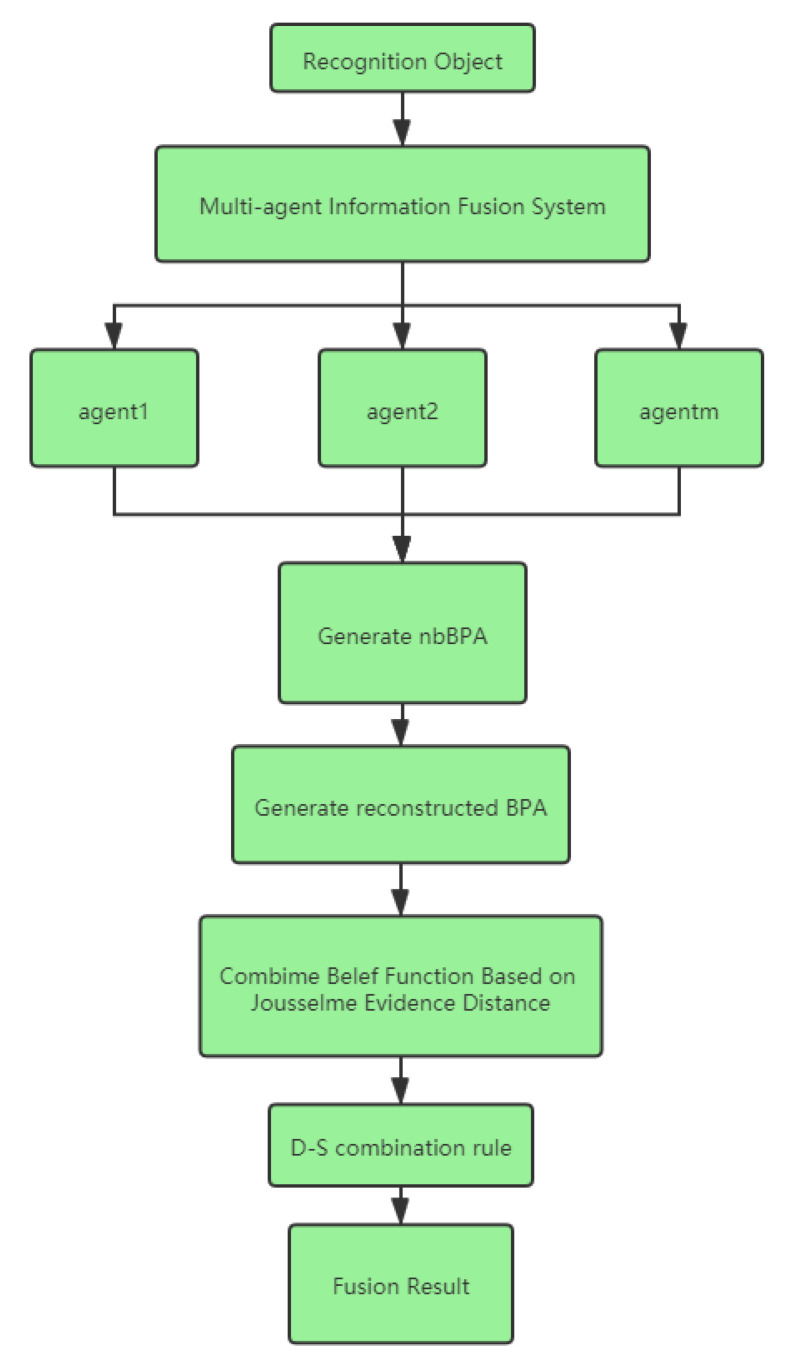
The proposed method for uncertain information fusion in the multi-agent information fusion system.

The method of the MAIF system is presented below, and the comprehensive processing flow chart is shown in Figure 1.

Step 1 The data of agent $A_{i}$ are converted into a mass function, and the form is $\left(A_{i},m_{i}\right)=(\left[\left\{s_{1}^{i}\right\},c_{1}^{i}\right]$, $\left[\left\{s_{2}^{i}\right\},c_{2}^{i}\right],\cdots ,\left[\left\{s_{2^{n}}^{i}\right\},c_{2^{n}}^{i}\right])$.
Step 2 nbBPA is generated by Equation (17), and it is combined with the original BPA to modify the BPA by Equation (18). Then, normalization is executed with Equation (19).
Step 3 The reconstructed BPA is generated according to Equation (15), and normalization is executed with Equation (16).
Step 4 The distance among different BPAs is measured with Jousselme evidence distance in Equation (8).
Step 5 Evidence modification based on Equations (10) and (14).
Step 6 Evidence combination with the D-S combination rule in Equation (4).

## 5. Numerical Examples

### 5.1. Example of Disturbed Agents

In real life [48,49,50], the agent may be disturbed when reading data information; thus, it may not work normally. One of the most common cases is that the interfered agents will have a high degree of conflict when making decisions [51] or reasoning [52] compared with that of other agents in the system. Therefore, the following example shows how to use the proposed method to effectively avoid such problems in the MAIF system. In the offshore operation, a group of multi-class sensor agents $A=\{A_{1},A_{2},A_{3},A_{4},A_{5}\}$ is used to identify the maritime target. An acoustic sensor agent, a speed sensor agent, a pressure sensitive sensor agent, and a photosensitive sensor agent are included. The FOD is Θ={A,B,C}. The data obtained by the corresponding agent are as follows:

$$\begin{array}{cc} A_{1}=\left\{\begin{array}{ccc} A & B & C\\ 0.5 & 0.2 & 0.3 \end{array}\right\} & A_{2}=\left\{\begin{array}{ccc} A & B & C\\ 0 & 0.9 & 0.1 \end{array}\right\}\\ A_{3}=\left\{\begin{array}{ccc} A & B & A,C\\ 0.55 & 0.1 & 0.35 \end{array}\right\} & A_{4}=\left\{\begin{array}{ccc} A & B & A,C\\ 0.55 & 0.1 & 0.35 \end{array}\right\}\\ A{}_{5}=\left\{\begin{array}{ccc} A & B & A,C\\ 0.6 & 0.1 & 0.3 \end{array}\right\} & \end{array}$$

The data monitored by agent $A_{2}$ are different from the data in other agents. This agent assigns most of the credibility to object B, while other agents assign higher reliability to object A. Therefore, the level of uncertainty among agents should be analyzed. The following is the main procedure to address this situation.

(1) The form of mass function is converted as follows:

$$\begin{array}{c} \left(A_{1},m_{1}\right)=\left(\left[\left\{A\right\},0.5\right],\left[\left\{B\right\},0.2\right],\left[\left\{C\right\},0.3\right]\right)\\ \left(A_{2},m_{2}\right)=\left(\left[\left\{A\right\},0\right],\left[\left\{B\right\},0.9\right],\left[\left\{C\right\},0.1\right]\right)\\ \left(A_{3},m_{3}\right)=\left(\left[\left\{A\right\},0.55\right],\left[\left\{B\right\},0.1\right],\left[\left\{A,C\right\},0.35\right]\right)\\ \left(A_{4},m_{4}\right)=\left(\left[\left\{A\right\},0.55\right],\left[\left\{B\right\},0.1\right],\left[\left\{A,C\right\},0.35\right]\right)\\ \left(A_{5},m_{5}\right)=\left(\left[\left\{A\right\},0.6\right],\left[\left\{B\right\},0.1\right],\left[\left\{A,C\right\},0.3\right]\right) \end{array}$$

(2) The nbBPA is generated, and the initial BPA is modified:

$$\begin{array}{ccc} m_{b}\left(A\right)=0.8 & m_{b}\left(B\right)=0.2 & m_{b}\left(C\right)=m_{b}\left(A,C\right)=0\\ m_{1}^{\prime }\left(A\right)=\frac{0.5+0.8}{2}=0.65 & m_{1}^{\prime }\left(B\right)=\frac{0.2+0.2}{2}=0.2 & m_{1}^{\prime }\left(C\right)=\frac{0.3+0}{2}=0.15\\ m_{2}^{\prime }\left(A\right)=0 & m_{2}^{\prime }\left(B\right)=\frac{0.9+0.2}{2}=0.55 & m_{2}^{\prime }\left(C\right)=\frac{0.1+0}{2}=0.05\\ m_{3}^{\prime }\left(A\right)=\frac{0.55+0.8}{2}=0.675 & m_{3}^{\prime }\left(B\right)=\frac{0.1+0.2}{2}=0.15 & m_{3}^{\prime }\left(A,C\right)=\frac{0.35+0}{2}=0.175\\ m_{4}^{\prime }\left(A\right)=\frac{0.55+0.8}{2}=0.675 & m_{4}^{\prime }\left(B\right)=\frac{0.1+0.2}{2}=0.15 & m_{4}^{\prime }\left(A,C\right)=\frac{0.35+0}{2}=0.175\\ m_{5}^{\prime }\left(A\right)=\frac{0.6+0.8}{2}=0.7 & m_{5}^{\prime }\left(B\right)=\frac{0.1+0.2}{2}=0.15 & m_{5}^{\prime }\left(A,C\right)=\frac{0.3+0}{2}=0.15 \end{array}$$

BPA is normalized:

$$\begin{array}{ccc} m_{1}^{\prime }\left(A\right)=0.65 & m_{1}^{\prime }\left(B\right)=0.2 & m_{1}^{\prime }\left(C\right)=0.15\\ m_{2}^{\prime }\left(A\right)=0 & m_{2}^{\prime }\left(B\right)=0.9167 & m_{2}^{\prime }\left(C\right)=0.0833\\ m_{3}^{\prime }\left(A\right)=0.675 & m_{3}^{\prime }\left(B\right)=0.15 & m_{3}^{\prime }\left(A,C\right)=0.175\\ m_{4}^{\prime }\left(A\right)=0.675 & m_{4}^{\prime }\left(B\right)=0.15 & m_{4}^{\prime }\left(A,C\right)=0.175\\ m_{5}^{\prime }\left(A\right)=0.7 & m_{5}^{\prime }\left(B\right)=0.15 & m_{5}^{\prime }\left(A,C\right)=0.15 \end{array}$$

(3) The original BPAs are reconstructed and normalized, and the results are shown as Table 1:

(4) The SMM is obtained by calculating the Jousselme evidence distance: 

$SMM=\left|\begin{array}{ccccc} 1 & 0.3142 & 0.8529 & 0.8529 & 0.8484\\ 0.3142 & 1 & 0.2145 & 0.2145 & 0.2107\\ 0.8529 & 0.2145 & 1 & 1 & 0.9928\\ 0.8529 & 0.2145 & 1 & 1 & 0.9928\\ 0.8484 & 0.2107 & 0.9928 & 0.9928 & 1 \end{array}\right|$

(5) The support and corresponding credibility degree are obtained by Equations (12) and (13).

$$\begin{array}{ccc} Sup(m_{1})=2.8685 & Sup(m_{2})=0.9540 & Sup(m_{3})=3.0603\\ Sup(m_{4})=3.0603 & Sup(m_{5})=3.0448 & \\ Crd_{1}=0.2209 & Crd_{2}=0.0735 & Crd_{3}=0.2356\\ Crd_{4}=0.2356 & Crd_{5}=0.2344 & \end{array}$$

(6) The credibility weights is assigned to the mass functions for MAE.

$$\begin{array}{cccc} m(A)=0.6956 & m(B)=0.2236 & m(C)=0.0393 & m(A,C)=0.0415 \end{array}$$

(7) Evidence is fused using the MAE four times, and the fusion result in the MAIF system is as follows:

$$\begin{array}{cccc} m(A)=0.9974 & m(B)=0.0026 & m(C)=0.00002 & m(A,C)=0.0000006 \end{array}$$

Table 2 shows the final data fusion results using different methods. From the final results, the test case has the highest trust in A. This is consistent with the actual situation and shows the rationality of the proposed method. In addition, the confidence level of the proposed method for A is 0.9974, which is higher than that of the original method by 0.9966. On this basis, the validity and rationality of the method are verified.

### 5.2. Example of Real-Time Processing

In some practical engineering applications, the objects identified from the system may change over time [53]. In this case, the MAIF system needs to collect information in real time and conduct corresponding decision analysis [54]. Supposing a military base has an MAIF system to identify targets, the system uses three agents to read real-time information. $A_{1}$ is an expert system agent, $A_{2}$ is a neural network agent, and $A_{3}$ is a GPS positioning agent. The FOD of the system refers to an aircraft, helicopter, and fighter. In order to correctly detect the type of dataset, the three agents must read this information in real time. Table 3 shows the details of the information read by MAS at three points in a certain period of time. Table 4 shows the results of different methods.

From Table 3, we can see that from the time $t_{1}$ to $t_{3}$, the probability of identifying the target as H gradually increases. H can be accurately identified at $t_{1}$. The fusion results show that the fusion effect of this method is consistent with the original method and has higher recognition accuracy.

### 5.3. Example of Setaria

In [55], Yuan et al. selected 120 samples as the training set and the remaining 30 samples as the test set to generate BPAs. Table 5 shows the BPAs of the four properties of the Setaria sample, where Θ represents the three species a,b,c.

If only the D-S combination rule is used, the fusion result may be illogical because of the zero value. Therefore, according to the method shown in Figure 1, all BPAs generated by the four attributes of the Setaria sample are corrected by using the nbBPA and reconstructed BPA. Then, the evidence distance is used to analyze the remaining steps of the method. The final combination results of different methods are shown in Table 6.

From the combination of the two methods, we can see that the BPA of proposition *a* is the highest. According to the final results, the samples are obviously *a*. In addition, in the BPA of the proposed method, *a* is higher than that in the original method. The experimental results verify the effectiveness and rationality of the method.

## 6. Conclusions

Due to the variability and interference of multi-source information, it is very important to consider the uncertainty relationship in the process of multi-source fusion. When processing information of multiple sources, it is easy to cause data conflicts due to various external factors, such as equipment damage. At the same time, the traditional D-S combination rule may produce counterintuitive results when addressing highly conflicting data. The nbBPA can be used as a method to solve this problem. nbBPA is based on the basic event. Other uncertain events in the power set of the FOD may not help in the process of decision making. Therefore, we only specify the initial base beliefs regarding the basic events. In this study, we improve the modeling of the uncertain relationship between the recognition objects in the MAIF system, and in this paper, an uncertainty model is established by adding nbBPA before reconstructing the BPA, combining it with the evidence distance factor and using the uncertainty relationship in evidence theory. The experimental results show that the method is effective and reasonable, especially when the multi-agent is faced with a highly unfavorable situation. This method can obtain correct results more accurately and quickly than other methods can, even in the case of equipment failure. Thus, it can be stated that this method improves the accuracy of the identification process of the MAIF system.

The following abbreviations are used in this manuscript:

## Figures and Tables

**Table 1:** The reconstructed and normalized BPAs.

	$m_{1}^{\prime }$	$m_{2}^{\prime }$	$m_{3}^{\prime }$	$m_{4}^{\prime }$	$m_{5}^{\prime }$
A	0.65	0	0.7788	0.7788	0.7895
B	0.2	0.9167	0.1593	0.1593	0.1579
C	0.15	0.0833	0	0	0
A,C	0	0	0.0619	0.0619	0.0526

**Table 2:** Fusion result of the example of disturbed agents.

Method	*m*(*A*)	*m*(*B*)	*m*(*C*)
D-S combination rule	0	0.1228	0.8772
Modified average combination rule of Deng et al. [43]	0.8909	0.0086	0.1005
Original method [25]	0.9966	0.0028	0.0005
Proposed method	0.9974	0.0026	0.00002

**Table 3:** Identification information read by agents.

	$t_{1}$	$t_{2}$	$t_{3}$
$A_{1}$	*m*(*A*) = 0.3666	*m*(*H*) = 0.8176	*m*(*H*) = 0.6229
	*m*(*H*) = 0.4563	*m*(*F*) = 0.0003	*m*(Θ) = 0.3771
	*m*(*A,H*) = 0.1185	*m*(*A,H*) = 0.1553	
	*m*((Θ) = 0.0586	*m*((Θ) = 0.0268	
$A_{2}$	*m*(*A*) = 0.2793	*m*(*H*) = 0.5658	*m*(*H*) = 0.7660
	*m*(*H*) = 0.4151	*m*(*F*) = 0.0009	*m*((*Θ)* = 0.2340
	*m*(*A,H*) = 0.2652	*m*(*A,H*) = 0.0646	
	*m*((Θ) = 0.0404	*m*((Θ) = 0.3687	
$A_{3}$	*m*(*A*) = 0.2897	*m*(*H*) = 0.2403	*m*(*H*) = 0.8598
	*m*(*H*) = 0.4331	*m*(*F*) = 0.0004	*m*(*(Θ)* = 0.1402
	*m*(*A,H*) = 0.2470	*m*(*A,H*) = 0.0141	
	*m*((Θ) = 0.0302	*m*((Θ) = 0.7452	

**Table 4:** Fusion results of the example of real-time processing.

	$t_{1}$	$t_{2}$	$t_{3}$
D-S combination rule	*m*(*A*) = 0.3376	*m*(*H*) = 0.9399	*m*(*H*) = 0.9876
	*m*(*H*) = 0.6317	*m*(*F*) = 0.0001	*m*(Θ) = 0.0124
	*m*(*A,H*) = 0.0305	*m*(*A,H*) = 0.0526	
	*m*(Θ) = 0.0001	*m*(Θ) = 0.0074	
Modified average combination rule of Deng et al.	*m*(*A*) = 0.3347	*m*(*H*) = 0.9065	*m*(*H*) = 0.9845
	*m*(*H*) = 0.6319	*m*(*F*) = 0.0002	*m*(Θ) = 0.0155
	*m*(*A,H*) = 0.0332	*m*(*A,H*) = 0.0403	
	*m*(Θ) = 0.0002	*m*(Θ) = 0.0530	
original method	*m*(*A*) = 0.3786	*m*(*H*) = 0.9451	*m*(*H*) = 0.9979
	*m*(*H*) = 0.6094	*m*(*F*) = 0.0189	*m*(Θ) = 0.0021
	*m*(*A,H*) = 0.0120	*m*(*A,H*) = 0.0298	
		*m*(Θ) = 0.0062	
proposed method	*m*(*A*) = 0.0250	*m*(*H*) = 0.9992	*m*(*H*) = 0.999992
	*m*(*H*) = 0.9749	*m*(*F*) = 0.0002	*m*(Θ) = 0.000008
	*m*(*A,H*) = 0.0018	*m*(*A,H*) = 0.0005	
	*m*(Θ) = 0.00000006	*m*(Θ) = 0.00003	

**Table 5:** $BPA_{S}$ of the four properties of the Setaria sample.

Attribute	m(a)	m(b)	m(a,b)	m(b,c)	m(a,b,c)
SL	0.2712	0	0	0	0.7288
SW	0	0.9	0	0.1	0
PL	0.6486	0	0	0	0.3514
PW	0.7477	0	0	0	0.2523

**Table 6:** Fusion result of the example of Setaria.

Attribute	m(a)	m(b)	m(a,b)	m(b,c)	m(a,b,c)
D-S combination rule	0	0.9	0	0.1	0
Modified average combination rule of Deng et al.	0.9133	0.0489	0	0.0037	0.0341
Original method	0.9997	0.0002	0	0	0.0001
Proposed method	0.999985	0.00001	0	0	0.000005

## Data Availability

Not applicable.
